# A decade of *G3: Genes|Genomes|Genetics*: a unified home for genetics and genomics research

**DOI:** 10.1093/g3journal/jkab247

**Published:** 2021-09-07

**Authors:** Brenda J Andrews

**Affiliations:** University of Toronto, Toronto, ON M5S 3E1, Canada

When we added *G3: Genes|Genomes|Genetics* to the Genetics Society of America’s publishing portfolio a decade ago, we discussed two compelling reasons for doing so (Andrews *et al.* 2011).

First, *GENETICS*, the flagship GSA journal, publishes papers that are of broad interest and provide novel—and often mechanistic—insight into genetic and genomic processes. This mandate, while important, meant that many useful studies in both established and emerging genetic systems were not being published by the GSA and thus made available to the community. For example, informal discussions with colleagues suggested that the results of interesting, well-designed genetic screens were languishing in lab notebooks as they did not yet have a clear mechanistic discovery to support a publication. However, access to those screen results could potentially catalyze work in other labs that would provide new insights. To address this gap, we created an article type called the Mutant Screen Report to make data from useful genetic screens available to the community in a timely fashion. Over the past 8 years, *G3* has published 118 Mutant Screen Reports, describing work in a range of experimental systems.

Second, a decade ago, it was clear that leaps in sequencing and other technologies were on the horizon. Space was needed for scientists to rapidly share important genomics data and resources that the community could build on as part of their research programs. To encourage dissemination of these data, *G3* added other article types like Genome Reports and Software and Database Resources. The Genome Report, in particular, has gained traction in the community as comparative genomics of both model and nonmodel systems has become possible with advances in genome editing and genome sequencing. In the past year, we have published Genome Reports reporting analyses of familiar model organism genomes as well as a remarkable range of biodiversity, from bacteriophages to birds, sea creatures, and plants.

The editorial mandate of *G3* was structured to complement and partner with our sister journal *GENETICS*, and to further our mission to ensure that practicing scientists play a central role in the peer-review process since the sharing of data, ideas, and conclusions is foundational to the practice of science. We began the journal with 60 editors and roughly 35% of our submissions coming to *G3* via *GENETICS*. Now, as we celebrate 10 years as an open-access publication, we have almost 100 associate editors led by 8 Senior Editors, a Deputy Editor, and the Editor in Chief. We now receive more than 85% of our submissions natively, while continuing to encourage interactions between *GENETICS and G3* editors to ensure that useful genetics and genomics data are made available to the community.

More than 3000 publications later, we are proud that our community believes in our mission and chooses to publish with us. And that mission? Serving the field of genetics by publishing high-quality, useful, and robust papers without subjective decisions about impact.

We believe that no journal should make the process of publishing a paper any harder than it needs to be. That’s why *G3* accepts first submissions in any format, lets you know promptly if your paper is not a good fit, and strives to return initial decisions for reviewed papers within 35 days. We accept preprints and participate in portable peer review (meaning you can bring reviews from another journal or review platform alongside your submission in hopes of expediting the review process). Our expert board of editors thoughtfully synthesize reviews and communicate clear decisions, so you’re not left wondering what to do next.

In our first 10 years, you’ve come to trust us as a place that treats you, your work, and your time with respect and care. A place that innovates and grows alongside the science and the scientists in our sphere. We hope to keep serving you with that same ethos for the next 10 years—and the 10 after that.

## Conflicts of interest

The author declare that there is no conflict of interest.
